# Successful Removal of Six Levonorgestrel Silastic Subcutaneous Implants (Norplant) 25 Years After Insertion: A Case Report

**DOI:** 10.7759/cureus.63585

**Published:** 2024-07-01

**Authors:** Evan A Old, Shilpa Gajarawala, Dacre R Knight, Kaitlyn Pak, Aakriti R Carrubba

**Affiliations:** 1 Department of Internal Medicine, Mayo Clinic, Jacksonville, USA; 2 Department of Medical and Surgical Gynecology, Mayo Clinic, Jacksonville, USA

**Keywords:** iatrogenic disease, levonorgestrel, contraceptive agents, contraception, norplant

## Abstract

Levonorgestrel-releasing silastic implants are a form of subdermal contraception that utilizes implanted silastic rods to release levonorgestrel, providing long-acting reversible contraception over an extended period of time. This case report presents a female who had lost a significant amount of weight after receiving levonorgestrel-releasing implants 25 years prior. During the elapsed period, the rods were palpable and uncomfortable. She had previously been unable to find a provider willing to remove the implants. This case highlights the possible complications surrounding the removal of levonorgestrel silastic subcutaneous implants and the careful consideration required when the implant has been in place for an extended period.

## Introduction

Norplant is a type of subdermal contraceptive that uses a levonorgestrel-releasing, silastic subdermal implant to prevent pregnancy; subdermal contraceptive implants fall under the category of long-acting reversible contraceptives. They offer high effectiveness and low failure rates [1, 2]. Norplant was first developed by the Population Council's International Committee for Contraceptive Research in 1966 and approved for use in the United States by the Food and Drug Administration in 1990 [3]. Each capsule contains 36 mg of dry crystalline levonorgestrel, for a total of 216 mg in six individual capsules [4]. This progestin diffuses into the surrounding tissues, where it is absorbed by the circulatory system on a continuous basis and provides a contraceptive effect for up to five years. The silastic rods measure 4 cm x 0.22 cm. The recommended placement location is subdermal in the medial aspect of the nondominant upper arm [5]. Due to issues with removal, Norplant was removed from the US market in 2002. The last insertions occurred in 2004 [6]. Removal of the implants should be completed upon patient request, for medical indications, or at the end of five years by clinicians educated on the proper removal technique [7]. Clinicians today may encounter patients with retained Norplant devices requesting removal. When removing such devices, careful attention should be paid to the initial placement location of the implant(s), time elapsed since implantation, and removal technique. Here we present a case that brings to light the complications of prolonged subcutaneous implants and a successful method of surgical extraction.

## Case presentation

**Figure 1:**
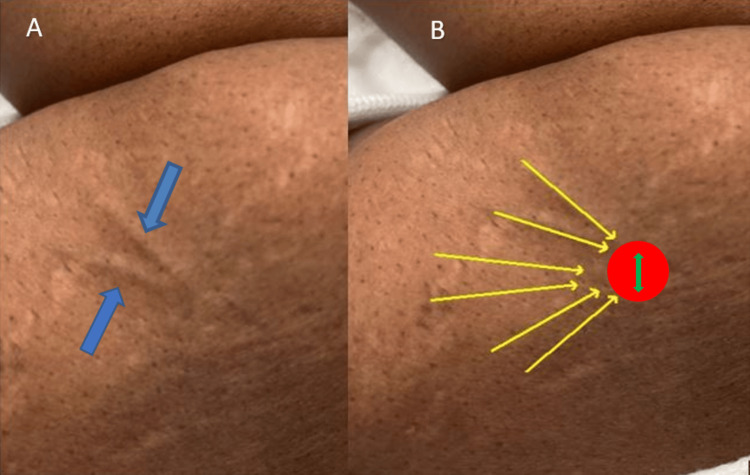
The figure shows (A) blue arrows pointing to the two implants visible to the naked eye, and (B) yellow arrows outlining the six total implants, all of which were palpable and noted to be intact upon investigation. The red circle depicts the confluence where lidocaine was injected, and the green arrow indicates the site of the 4 mm incision made by #11 scalpel.

**Figure 2:**
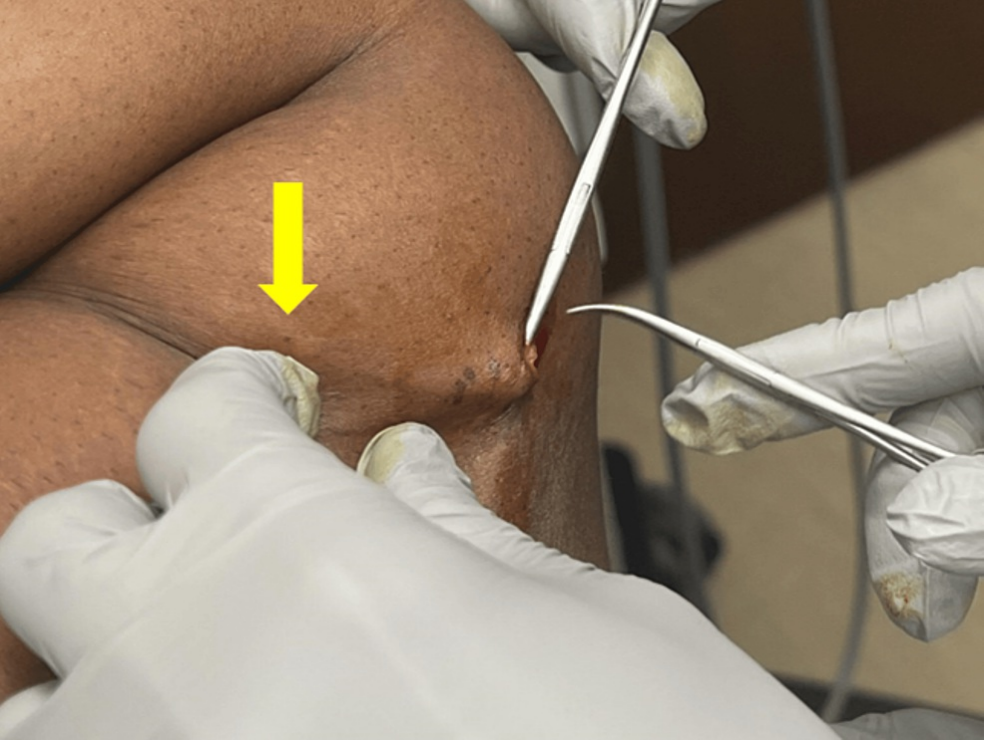
Manual pressure being applied at the proximal end of the implant (yellow arrow), pushing the distal tip encased in a fibrous sheath toward the incision

**Figure 3:**
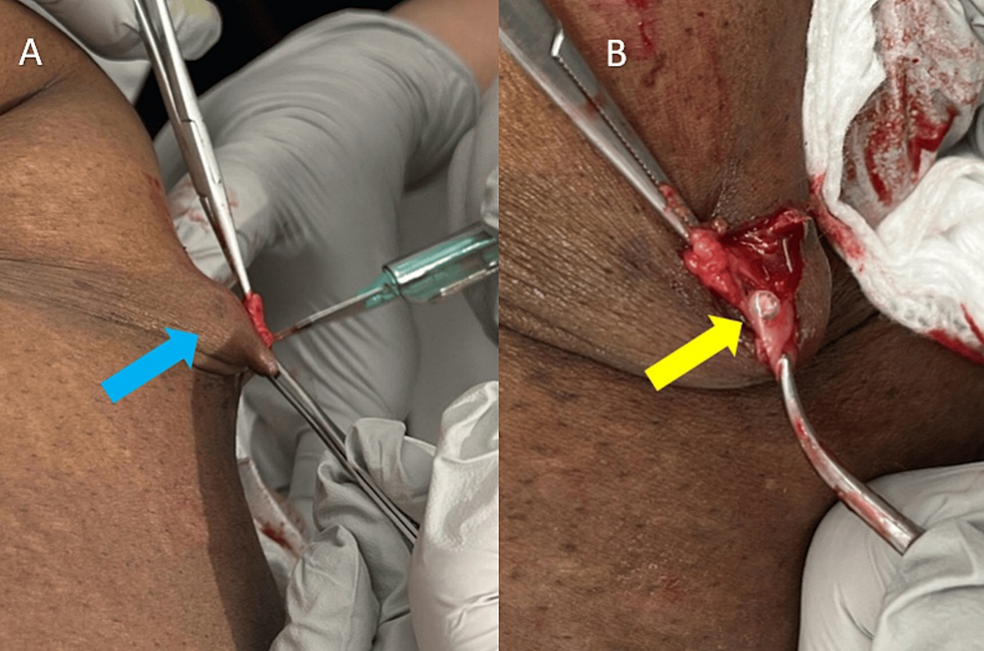
(A) the fibrous sheath being incised (blue arrow) until the implant becomes visible; (B) the visible implant (yellow arrow) at the incision site.

**Figure 4:**
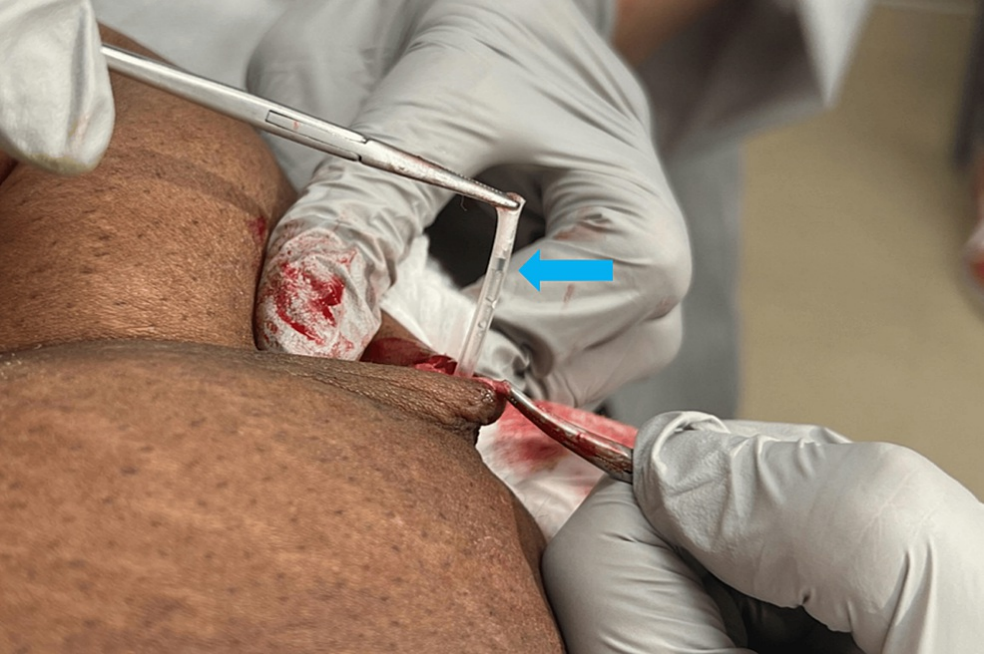
The implant (blue arrow) being grasped and removed with forceps. In total, six 4-cm levonorgestrel silastic subcutaneous implants were removed intact through the singular incision.

**Figure 5:**
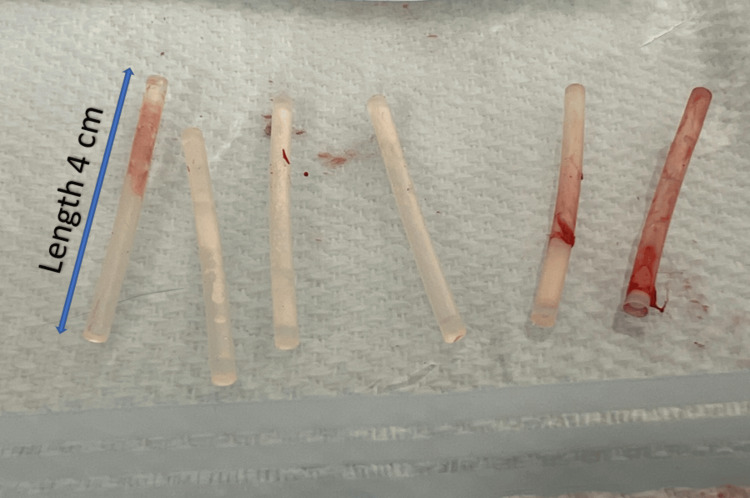
All six 4 cm levonorgestrel silastic subcutaneous implants were intact following removal through the initial 4 mm incision (blue arrow), showing the length (4 cm) of the extracted levonorgestrel silastic subcutaneous implants.

A 51-year-old female presented to a gynecology practice for a Norplant removal 25 years after placement. She had lost a significant amount of weight during this time. She reported that the rods were palpable and uncomfortable. She had previously been unable to find a trained provider willing to remove the implants. Upon initial inspection, only two rods were visible (Figure 1A), but all six were palpable (Figure 1B). The risks and benefits of removal were discussed, and the patient provided informed consent to proceed with office removal. Labs were not drawn prior to implant removal. Lidocaine 1% (2 mL) was injected at the confluence (Figure 1B), and lidocaine 1% (.5 mL) was injected under each rod in a tumescent manner to displace the implants superficially and numb the surrounding area; no further medications were administered during or after the procedure. A 4 mm incision (Figure 1B) was made with a #11 scalpel. Using pressure at the proximal end of the implant (Figure 2), the distal tip, encased in a fibrous sheath, was pushed toward the incision. The fibrous sheath was incised (Figure 3A) until the implant became visible (Figure 3B). The implant (Figure 4) was grasped and removed with forceps. All six 4 cm levonorgestrel implants were removed intact in this manner through the same incision (Figure 5). The incision was closed with one subcuticular stitch and a pressure dressing. There were no complications during or after the procedure. 

## Discussion

Clinicians today may encounter patients with retained Norplant devices requesting removal. Prolonged implantation may cause complications upon the removal of these devices. In these cases, special consideration should be given to possible complications involving device migration and the depth of the device [8]. 

As with many surgical procedures, there are a variety of published Norplant removal techniques [9-11]. No one procedure appears to have a universal advantage over others; rather, removal procedures should be guided by the patient's medical history and exam. Removal of the implant rods may be challenging; thus, adequate time and careful technique are necessary. A video demonstration of the surgical technique for this case was shared at the Society of Gynecologic Surgeons 49^th^ Annual Scientific Meeting in March 2023 [12]. The basis for successful administration and removal is a correct subdermal insertion at the start. To avoid infections and excessive scarring during excision, special attention should be given to the following: aseptic technique, correct subdermal placement of the rods, and minimization of tissue trauma [9]. If the rods are placed deeply, they are more challenging to remove. The silastic rods may, at times, be difficult to locate and are sometimes fractured, nicked, cut, or broken [8]. Instances of traumatic ulnar nerve injury have been recorded during the removal of improperly implanted Norplant devices [13-14]. Based on a study of 849 removals over a five-year period, with a frequency of 6.2%, removal difficulties include the need for multiple incisions, rod fragmentation and retention, pain, the need for multiple visits, deep placement, lengthy procedure, or neurovascular injury [8]. Before initiating removal, all rods should be located by palpation. If all six rods cannot be located by palpation, they may be localized by ultrasound, X-ray, compression mammography, computed tomography (CT), or magnetic resonance imaging (MRI) [12-13]. In-office attempts to remove contraceptive implants that are deep or have migrated can cause iatrogenic injury to the median, ulnar, and medial antebrachial cutaneous nerves [14]. 

Patients with neurologic symptoms after placement or removal attempts require prompt investigation and referral to neurological specialists. If the removal of the rods proves difficult, it is advised to interrupt the procedure and offer a return visit, as the remaining rod(s) will be easier to remove once healing in the area matures. It is appropriate to seek consultation or provide referral to dermatologic or surgical subspecialists for patients in whom initial attempts at rod removal prove difficult.

## Conclusions

This case demonstrates an emerging complication of subdermal contraceptive implants associated with prolonged implantation. Norplant implants were removed from the US market in 2002; thus, current patients with these implants will likely face similar complications of removal. The most effective removal technique is also the safest and requires careful medical history taking and a physical exam, possibly with the addition of advanced imaging and a subspecialty referral. Our report shows the importance of recognizing the complications of prolonged Norplant implantation and the best technique for removal. Future research should systematically assess techniques for Norplant removal.
